# Repeatability of tumour hypoxia imaging using [^18^F]EF5 PET/CT in head and neck cancer

**DOI:** 10.1007/s00259-017-3857-3

**Published:** 2017-10-26

**Authors:** Antti Silvoniemi, Sami Suilamo, Timo Laitinen, Sarita Forsback, Eliisa Löyttyniemi, Samuli Vaittinen, Virva Saunavaara, Olof Solin, Tove J. Grönroos, Heikki Minn

**Affiliations:** 1 Turku PET Centre, University of Turku, P.O. BOX 52, FI-20521 Turku, Finland; 2 Department of Otorhinolaryngology – Head and Neck Surgery, Turku University Hospital, P.O. BOX 52, FI-20521 Turku, Finland; 3 Department of Oncology and Radiotherapy, Turku University Hospital, P.O. BOX 52, FI-20521 Turku, Finland; 4 Department of Medical Physics, Turku University Hospital, P.O. BOX 52, FI-20521 Turku, Finland; 5 Department of Biostatistics, University of Turku, Turku, Finland; 6 Department of Pathology, Turku University Hospital, P.O. BOX 52, FI-20521 Turku, Finland

**Keywords:** Repeatability, Hypoxia, Pet, Head and neck cancer, ^18^F-EF5

## Abstract

**Purpose:**

Hypoxia contributes to radiotherapy resistance and more aggressive behaviour of several types of cancer. This study was designed to evaluate the repeatability of intratumour uptake of the hypoxia tracer [^18^F]EF5 in paired PET/CT scans.

**Methods:**

Ten patients with newly diagnosed head and neck cancer (HNC) received three static PET/CT scans before chemoradiotherapy: two with [^18^F]EF5 a median of 7 days apart and one with [^18^F]FDG. Metabolically active primary tumour volumes were defined in [^18^F]FDG images and transferred to co-registered [^18^F]EF5 images for repeatability analysis. A tumour-to-muscle uptake ratio (TMR) of 1.5 at 3 h from injection of [^18^F]EF5 was used as a threshold representing hypoxic tissue.

**Results:**

In 10 paired [^18^F]EF5 PET/CT image sets, SUVmean, SUVmax, and TMR showed a good correlation with the intraclass correlation coefficients of 0.81, 0.85, and 0.87, respectively. The relative coefficients of repeatability for these parameters were 15%, 17%, and 10%, respectively. Fractional hypoxic volumes of the tumours in the repeated scans had a high correlation using the Spearman rank correlation test (*r* = 0.94). In a voxel-by-voxel TMR analysis between the repeated scans, the mean of Pearson correlation coefficients of individual patients was 0.65. The mean (± SD) difference of TMR in the pooled data set was 0.03 ± 0.20.

**Conclusion:**

Pretreatment [^18^F]EF5 PET/CT within one week shows high repeatability and is feasible for the guiding of hypoxia-targeted treatment interventions in HNC.

## Introduction

Hypoxia is among the strongest biological factors causing radiotherapy (RT) resistance in several types of cancer [1]. The outcome of RT in head and neck cancer (HNC) is particularly sensitive to the oxygenation status where numerous studies show a worse prognosis associated with tumour hypoxia [2, 3]. To overcome radiation resistance, dose-escalation protocols have been developed where hypoxic subvolumes receive higher doses based on PET/CT imaging with tracers preferentially accumulating in poorly oxygenated tissues [4]. One of these PET tracers is a fluorine-18 labelled form of 2-(2-nitro-1H-imidazol-1-yl)-N-(2,2,3,3,3-pentafluoropropyl)-acetamide (EF5), which has been thoroughly evaluated for its hypoxia-avidity potential both in vitro and in vivo [5, 6]. Our previous studies have shown favourable tumour uptake characteristics [7] and a prognostic value of [^18^F]EF5 PET/CT imaging in patients with HNC [8].

The repeatability of hypoxia PET imaging is crucial for feasibility in clinical applications such as the planning of RT. A few clinical studies have been conducted previously using voxel-by-voxel analysis of spatial tracer distribution in HNC and lung cancer in paired scans within a short pretreatment period with [^18^F]FMISO PET/CT [9–11] and [^18^F]HX4 PET/CT [12]. Three of these studies reported highly repeatable results of hypoxia PET imaging [10–12], while a single study showed a lower linear correlation than the other studies between the intratumour tracer uptake in the repeated scans [9]. These studies were performed using a fixed interval of two [10] or three [9] days or an average interval of 1–2 days [11, 12], respectively.

Owing to the potential differences in biodistribution and imaging characteristics of the available hypoxia-avid PET tracers, it is important to investigate their repeatability individually in the test-retest setting. Therefore, we aimed to measure the repeatability of [^18^F]EF5 PET/CT imaging in HNC before the start of RT and focus on the possibility of comparing our findings to [^18^F]FMISO and [^18^F]HX4, which have been evaluated in comparable studies and patients.

## Patients and methods

### Patients

This prospective study (NCT 01774760) was conducted at Turku University Hospital, Finland between September 2013 and September 2016. Patients between 18 and 80 years of age with untreated pharyngeal squamous cell carcinoma referred to definitive chemoradiotherapy (CRT) were eligible and were required to have a WHO performance status 0-2, without a history of previous head and neck malignancies or RT in the head and neck area. Additional exclusion criteria were serious cardiac, pulmonary, renal, liver and haematological disorders, pregnancy, or nursing. Eleven patients signed the consent form and underwent all study procedures, but one tonsillar cancer patient was excluded from analyses due to an extremely low tracer uptake in the primary tumour in pretreatment [^18^F]FDG PET/CT imaging after diagnostic tonsillectomy, which preceded PET/CT imaging. The characteristics of the remaining 10 patients are presented in Table 1. Four of them had primary tumour positive for p16 which has been linked to human papilloma virus (HPV) infection [13].

**Table 1:** Patient characteristics. All patients in this study were men

Patient nr	Age (years)	Weight (kg)	Tumour site	TNM	Stage	HPV status^a^	GTV^b^ (cm^3^)
1	73	77	Base of tongue	T2N1M0	III	+	8.9
2	68	49	Base of tongue	T3N0M0	III	–	40.3
3	56	86	Base of tongue	T3N1M0	III	+	51.9
4	69	66	Oropharyngeal wall	T4aN1M0	IVa	–	17.0
5	58	125	Base of tongue	T2N2cM0	IVa	–	20.5
6	60	84	Nasopharynx	T4N3bM0	IVb	+	94.0
7	69	94	Hypopharynx	T4bN2cM0	IVb	–	52.4
8	76	78	Nasopharynx	T4N2bM0	IVa	–	73.5
9	66	77	Tonsil	T4aN2cM0	IVa	+	30.4
10	66	85	Hypopharynx	T4aN2bM0	IVa	–	25.4

^a^ Obtained from immunohistochemical analysis for presence of cyclin dependent kinase inhibitor 2A (p16) in tumour cells

^b^ GTV = gross tumour volume manually delineated in CT image

### Synthesis of [^18^F]EF5

[^18^F]EF5 was synthesised as previously described [14]. The molar activity of [^18^F]EF5 decay corrected to the end of synthesis exceeded 8 GBq/μmol. Radiochemical purity was higher than 98.5% in every production batch.

### Imaging protocol

All patients underwent two [^18^F]EF5 PET/CT acquisitions with a median interval of 7 days (range 5–7 days). The first (EF5#1) and the second (EF5#2) scan were performed with identical acquisition protocols using the same scanner GE D690 PET/CT (General Electric Medical Systems, Milwaukee, WI, USA). Before each of the paired acquisitions, the patients received an intravenous mean (± SD) dose of [^18^F]EF5 of 303 ± 23 MBq (range 246–345 MBq). The mean (±SD) intrapatient difference between injected doses was 18 ± 12 MBq (range 1–40 MBq). A low-dose CT (120 kV, noise index 20, Asir 40%) for anatomical reference and attenuation correction was obtained immediately before PET acquisition, which started 178 ± 9 min post injection (range 160–190 min). The PET acquisition time was 6 min covering an axial field of view (FOV) of 15 cm with a slice thickness of 3.27 mm. The intrapatient difference between the start of the acquisition time of repeated [^18^F]EF5 PET/CT scans was 7 ± 6 min (range 0–19 min). The patients were immobilised on the flat scanner table using a thermoplastic mask. Venous blood samples were taken before and after the imaging session, and blood activity at the mid-point of image acquisition was calculated using linear interpolation with decay-corrected blood activity values.

On a separate day, all patients underwent whole-body ^18^F-fluorodeoxyglucose (FDG) PET/CT imaging following the standard institutional protocol used in RT planning [15]. The sequential scans were performed in random order with [^18^F]FDG PET/CT either between or after the two [^18^F]EF5 PET/CTs. [^18^F]FDG PET/CT was performed with the same GE D690 scanner as the hypoxia scans, except for patients nr 1, 2, 5, and 7, who were imaged with the Discovery VCT PET/CT scanner (General Electric Medical Systems, Milwaukee, WI, USA).

The GE D690 PET/CT scanner images were reconstructed using a 192 × 192 matrix with a transaxial FOV of 70 cm. In order to achieve a uniform voxel size (3.65 × 3.65 × 3.27 mm) for all PET images, a corresponding 128 × 128 reconstruction matrix with a transaxial FOV of 46.7 cm was selected for the GE Discovery VCT PET/CT.

### Image analysis

Varian Eclipse software version 13.6 (Varian Medical Systems, Palo Alto, CA, USA) was used for the determination of tracer uptake in primary tumour and reference tissue in all PET images. The delineation of the primary tumour volume of interest (VOI) was based on the metabolically active tumour volume (MATV) in the [^18^F]FDG image using either a threshold of 40% of SUVmax or a fixed SUV 5.0 threshold, depending on which more closely matched the CT-based anatomical gross tumour volume (GTV). Posterior neck muscles were used as reference tissue for tracer uptake [7]. The [^18^F]FDG and [^18^F]EF5 images were rigidly registered using anatomical information from CT images.

Carimas 2.9 software (www.turkupetcentre.fi/carimas) was used for voxel-by-voxel analysis of [^18^F]EF5 PET/CT images. The transformation matrices were applied to the [^18^F]EF5 images to define the MATV-based primary tumour VOIs in [^18^F]EF5 images. The accuracy of VOI structure transformations for voxel-by-voxel analyses was controlled using visual inspection of images and cross tabulation of tumour uptake values.

The uptake was measured as kBq/mL and then decay corrected and converted to standardised uptake values (SUV) under the assumption of water density. Tumour SUVmean and SUVmax uptakes were determined, as well as mean uptake in posterior neck muscle reference (SUVmuscle). Hypoxic subvolume of the tumour (HV) was determined using a tumour-to-muscle uptake ratio (TMR) of 1.5 as a threshold for hypoxia [7]. Fractional hypoxic volume (FHV) was calculated by dividing the number of hypoxic voxels with the total number of voxels within tumour VOI.

### Statistical analysis

Data expressed with plus/minus indicates mean and standard deviation (SD). A paired T-test was used for comparison of injected doses, injected doses per weight, and acquisition starting times of individual patients between EF5#1 and EF5#2. Correlations of normally distributed tumour-level parameters were assessed by calculating intraclass correlation coefficients (ICC). For FHVs and HVs, a non-parametric Spearman rank correlation test was used. Pearson correlation coefficients were calculated for repeated voxel-level uptake parameters. Bland-Altman plots were constructed for agreement analysis of both the tumour- and voxel-level parameters. In addition, upper and lower limits of agreement (LoA) and coefficient of repeatability (CoR) were calculated. *p* < 0.05 was used as a level of significance (two-tailed). For the test of normality, the Shapiro-Wilk test was used for tumour-level parameters and visual assessment for voxel-level parameters. The statistical analyses were performed using SAS software version 9.4 (SAS institute, Cary, NC, USA).

## Results

**Fig. 1:**
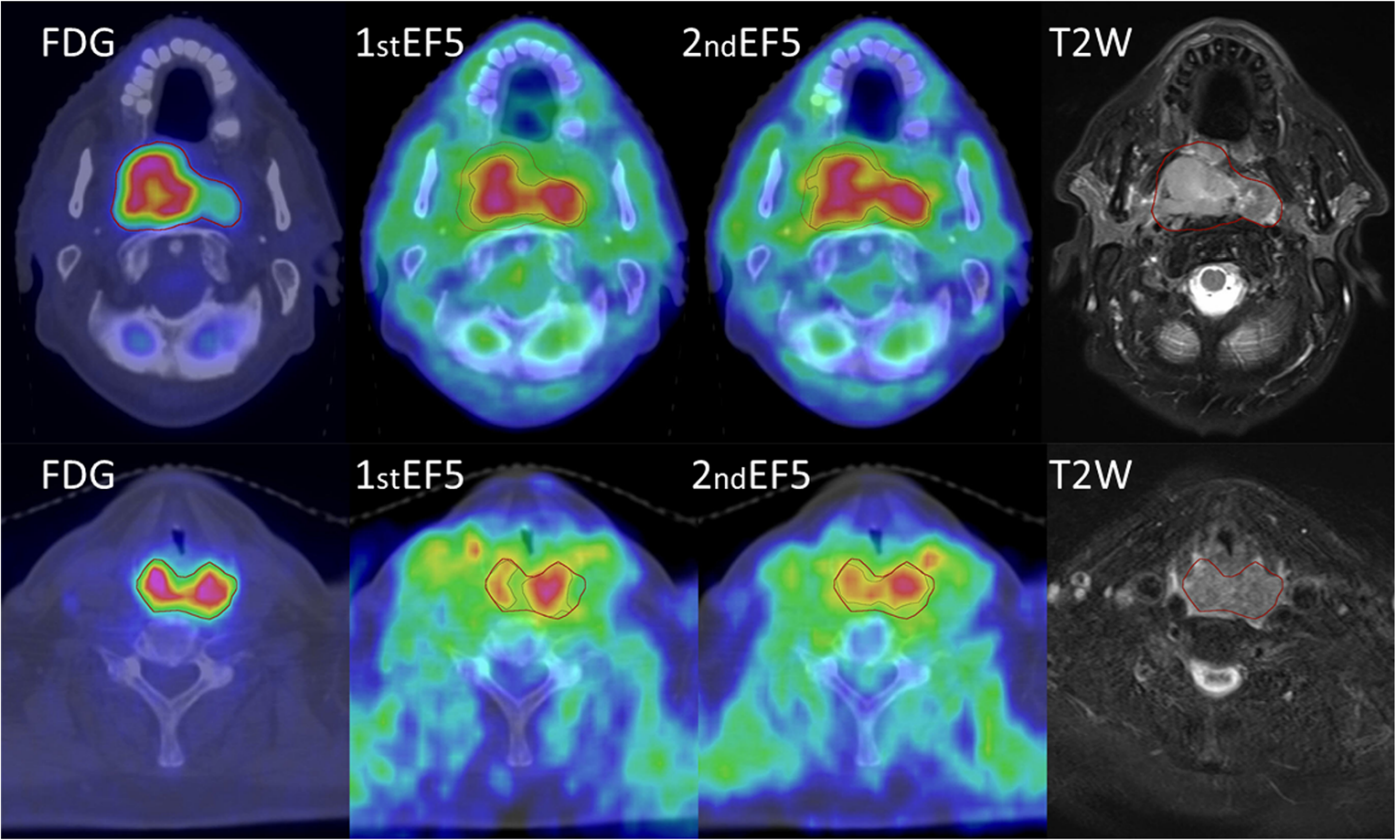
PET/CT and MR images of patients presenting with nasopharyngeal cancer (No. 6; upper row) and hypopharyngeal cancer (No. 7; lower row). From left to right, corresponding axial slices from diagnostic [^18^F]FDG, the first and the second [^18^F]EF5 PET/CT; and fat-suppressed T2-weighted MR images are shown. The red line denotes the metabolically active tumour volume delineation using SUV 5.0 as a threshold in the [^18^F]FDG PET image. The black line indicates hypoxic subvolume delineation using a tumour-to-muscle uptake ratio of 1.5 as a threshold in the [^18^F]EF5 PET image. The intrapatient voxel-by-voxel analysis showed a high correlation and agreement between the paired [^18^F]EF5 PET/CT images for patient No. 6, while those for patient No. 7 were among the lowest of 10 patients (see results of individual patients in Fig. 3 and Tables 2 and 3)

Anatomical GTVs of the primary tumours measured in the CT images varied considerably. The average GTV was 41.4 ± 26.9 cm^3^ (range 8.9–94 cm^3^) and the corresponding MATV was 39.0 ± 26.7 cm^3^ (range 7.2–100 cm^3^). There were no statistically significant differences between injected doses, injected doses per weight, and scanning start times of individual patients within repeated [^18^F]EF5 PET/CT scans (for all comparisons *p* > 0.36). Examples of two paired [^18^F]EF5 PET/CT images are shown in Fig. 1.

**Fig. 2:**
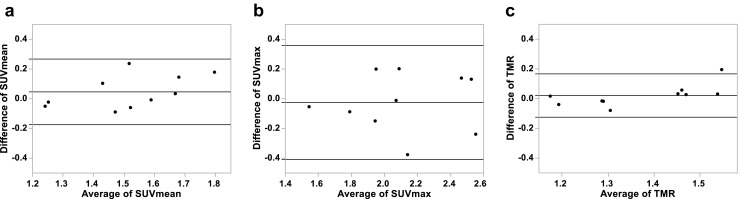
Bland-Altman plots of SUVmean (a), SUVmax (b), and tumour-to-muscle uptake ratio (TMR) (c) of repeated [^18^F]EF5 PET/CT scans. Beginning from the most superior one, the three solid lines represent the upper limit of agreement (LoA), the mean difference, and the lower LoA, respectively

The average whole tumour uptake of [^18^F]EF5 (SUVmean) among all patients was at the same level in the first scan (1.49 ± 0.16) and in the second scan (1.54 ± 0.21). The corresponding values for the highest uptake (SUVmax) were 2.12 ± 0.34 and 2.09 ± 0.35, respectively (Table 2). The correlation of these parameters within individual patients in repeated scans was high. The ICCs were 0.81 (*p* < 0.001) for SUVmean and 0.85 (*p* < 0.001) for SUVmax. The mean differences of SUVmean and SUVmax were 0.05 ± 0.11 and −0.02 ± 0.20, respectively. Bland-Altman plots of these parameters are presented in Fig. 2. The relative CoRs for SUVmean and SUVmax were 15% and 17%, respectively.

**Table 2:** Tumour-level and muscle uptake parameters in the repeated [^18^F]EF5 PET/CT scans

Patient nr	SUV_mean_ Scan 1	SUV_mean_ Scan2	SUV_max_ Scan 1	SUV_max_ Scan2	TMR Scan 1	TMR Scan 2	FHV% Scan 1	FHV% Scan 2	HV Scan 1 (cm^3^)	HV Scan 2 (cm^3^)	SUVmuscle Scan 1	SUVmuscle Scan 2
1	1.61	1.75	1.99	2.19	1.43	1.49	21.2	34.5	1.5	2.5	1.12	1.18
2	1.26	1.24	1.57	1.52	1.30	1.28	3.8	1.2	1.3	0.4	0.97	0.97
3	1.59	1.58	2.08	2.06	1.29	1.28	6.8	3.5	3.4	1.8	1.23	1.24
4	1.27	1.22	1.83	1.75	1.17	1.18	4.9	4.3	1.0	0.9	1.09	1.03
5	1.55	1.49	2.33	1.95	1.34	1.27	19.1	6.7	4.0	1.4	1.15	1.18
6	1.65	1.68	2.46	2.59	1.52	1.55	40.5	45.2	40.6	45.3	1.08	1.09
7	1.40	1.64	2.67	2.43	1.46	1.48	34.2	40.3	18.3	21.6	0.96	1.10
8	1.71	1.89	2.40	2.53	1.45	1.64	32.6	63.7	16.4	32.0	1.18	1.15
9	1.38	1.48	1.85	2.05	1.43	1.47	27.8	32.4	10.4	12.1	0.96	1.01
10	1.52	1.43	2.02	1.87	1.21	1.17	1.5	0.3	0.3	0.0	1.25	1.22
Mean	1.49	1.54	2.12	2.09	1.36	1.38	19.3	23.2	9.7	11.8	1.10	1.12
SD	0.16	0.21	0.34	0.35	0.12	0.16	14.3	22.8	12.7	16.0	0.11	0.09

*TMR* tumour-to-muscle uptake ratio, *FHV* fractional hypoxic volume, *HV* hypoxic volume

SUVmuscle was stable within individual patients between the repeated scans (Table 2). The ICC for SUVmuscle was 0.84 (*p* < 0.001) and the mean difference of SUVmuscle between the scans was 0.15 ± 0.06 with the upper and lower LoA of 0.26 and 0.04, respectively. The relative CoR for SUVmuscle was 10%. Additionally, activity in venous blood samples measured as SUV showed a high correlation and agreement between the scans, with ICC being 0.94 (*p* < 0.001) and relative CoR 10%.

The highest correlation and agreement among tumour-level uptake parameters were observed within those of TMR. The ICC for TMR was 0.87 (*p* < 0.001) and the mean difference was 0.02 ± 0.07, with the upper and lower LoA of 0.17 and −0.12, respectively (Fig. 2). The relative CoR for TMR was 10%. The parameters of HV and FHV did not show normal distribution, and therefore the correlation of these parameters between repeated scans was assessed with a Spearman correlation test. A high and statistically significant correlation was observed, both for repeated HV (*r* = 0.93, *p* < 0.001) and FHV (*r* = 0.94, *p* < 0.001).

**Fig. 3:**
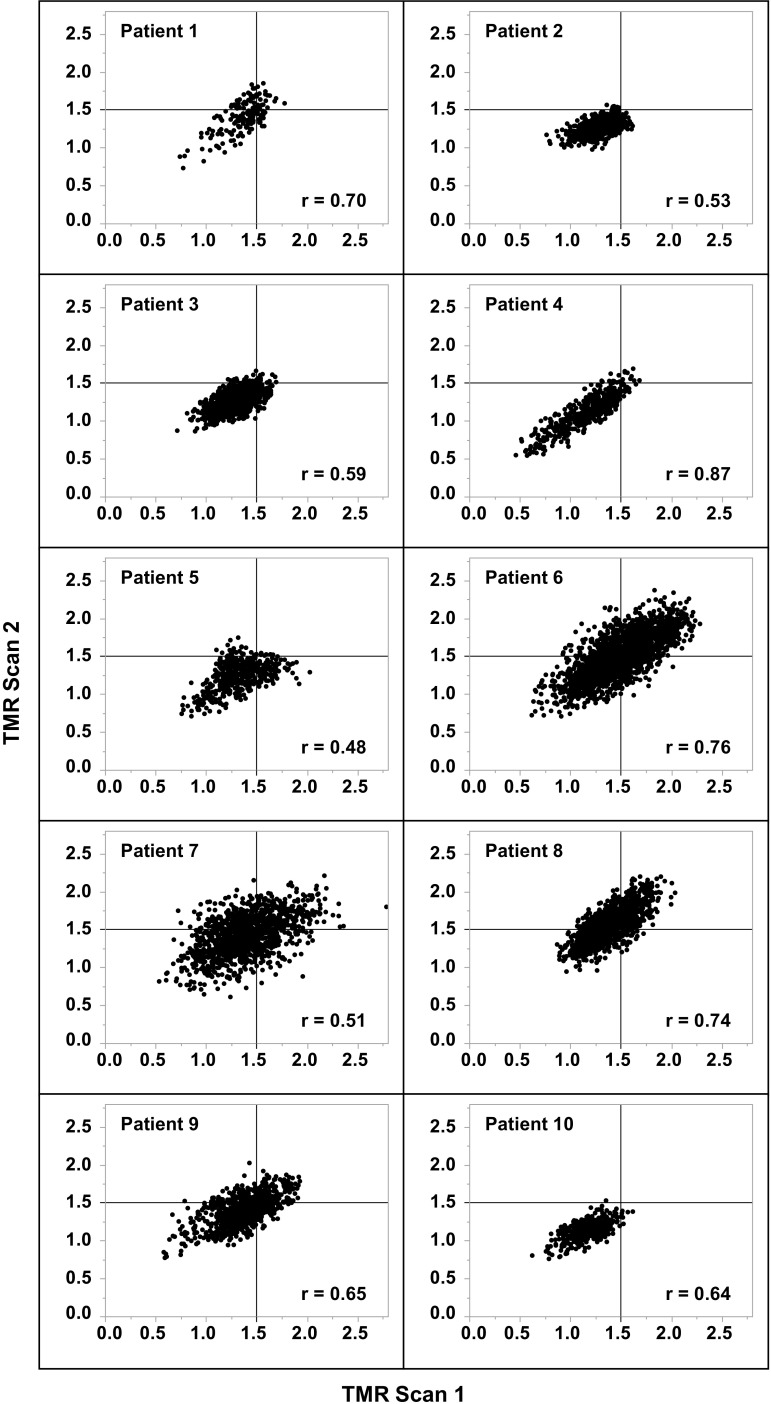
Scatterplots of voxelwise tumour-to-muscle uptake ratios (TMR). The X-axis represents the first and the Y-axis the second of the [^18^F]EF5 PET/CT scans. Solid lines indicate the cutoff level for hypoxia (TMR 1.5)

**Fig. 4:**
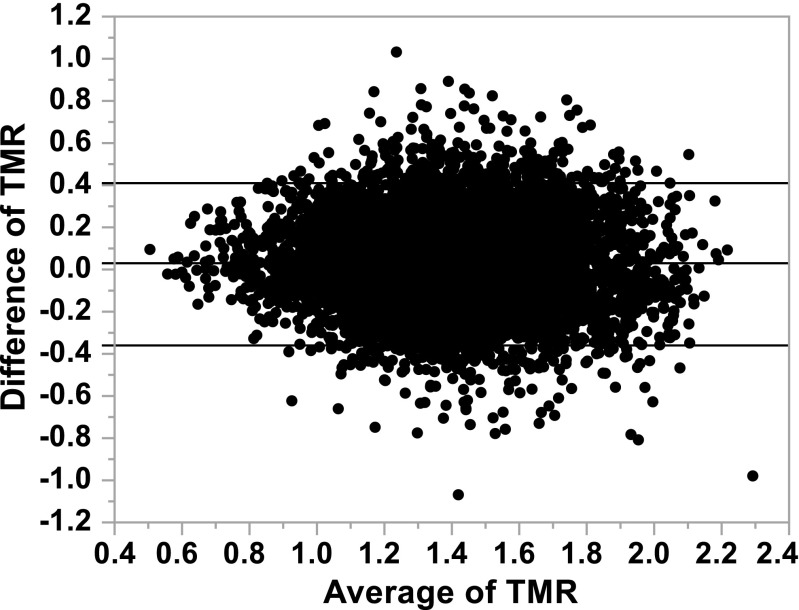
A Bland-Altman plot of voxelwise tumour-to-muscle uptake ratios (TMRs) from the pooled data of all patients. Beginning from the most superior one, the three solid lines represent the upper limit of agreement (LoA), the mean difference, and the lower LoA, respectively

In the voxel-by-voxel analysis, the mean of Pearson correlation coefficients between TMR of [^18^F]EF5 in the repeated scans within individual patients was 0.65 (range 0.48–0.87). The scatterplots of individual patients are presented in Fig. 3. The mean differences of voxel-level TMRs of individual patients between the paired scans with upper and lower LoA are shown in Table 3. The mean calculated from mean differences of individual patients was 0.02 ± 0.07. For the pooled dataset, the mean difference of voxelwise TMR was 0.03 ± 0.20, with an upper and lower LoA of 0.41 and −0.36, respectively (Fig. 4), and the absolute CoR and relative CoR were 0.39 and 28%, respectively.

**Table 3:** Results of voxel-level agreement analysis between tumour-to-muscle uptake ratios of repeated [^18^F]EF5 PET/CT scans

Patient nr	Number of voxels	Mean ± SD difference (95% CI)	Upper LoA	Lower LoA
1	165	0.05 ± 0.16 (0.02 — 0.07)	0.36	−0.26
2	757	0.00 ± 0.12 (−0.01 — 0.01)	0.23	−0.23
3	1159	−0.02 ± 0.13 (−0.02 — -0.01)	0.23	−0.26
4	470	−0.03 ± 0.12 (−0.04 — -0.02)	0.21	−0.27
5	476	−0.07 ± 0.21 (−0.09 — -0.05)	0.34	−0.48
6	2306	0.02 ± 0.20 (0.01 — 0.02)	0.40	−0.37
7	1233	0.03 ± 0.28 (0.02 — 0.05)	0.59	−0.52
8	1155	0.18 ± 0.16 (0.17 — 0.19)	0.48	−0.13
9	860	0.03 ± 0.18 (0.02 — 0.04)	0.39	−0.33
10	394	−0.02 ± 0.12 (−0.03 — 0.00)	0.22	−0.26
Pooled dataset	8975	0.03 ± 0.20 (0.02 — 0.03)	0.41	−0.36

*CI* confidence interval, *LoA* limit of agreement

## Discussion

This study was designed to assess the repeatability of [^18^F]EF5 PET/CT among HNC patients before the onset of definitive CRT. For each individual oncologic PET tracer, a test-retest study is a fundamental part of the feasibility evaluation for clinical applications [16]. However, from a methodological point of view, the comparison of repeated hypoxia PET scans has been considered difficult to be reduced into a robust assessment of accuracy in quantitative analysis since the measured phenomenon has been assumed to change over the short term due to the so-called cycling hypoxia [17]. Thus, the results of hypoxia PET repeatability studies have been interpreted as a combination of the technical repeatability of measurements and true changes in tumour oxygenation. Nevertheless, at the resolution reported here, which is entirely adequate to apply image-guided targeted therapy, cycling hypoxia does not appear to be a significant factor.

In this study, the [^18^F]EF5 uptake in primary HNC was comparable to those reported previously [7, 8]. The definition of tumour VOI for repeatability assessment was based on MATV in the [^18^F]FDG image to facilitate comparison with some of the previous clinical hypoxia PET repeatability studies [9, 11]. For this purpose, we prefer MATV to pure CT-based delineation in order to avoid the inclusion of background tissues, which might cause an erroneously high repeatability of intratumour [^18^F]EF5 uptake [18, 19]. This is mainly due to the presumption that tissues in the periphery with low [^18^F]FDG uptake would be expected to show less dynamic and more repeatable hypoxia tracer uptake than the core of the tumour. Posterior neck muscles were used as reference tissue and the uptake of [^18^F]EF5 in muscle within paired scans was observed to have a good correlation (ICC 0.84) and high repeatability with a relative CoR of 10%. Furthermore, the findings in venous blood samples supported the assumption of stable radioactivity concentration in the blood pool, and thus, highly repeatable activity measurements.

A TMR of 1.5 was derived as a threshold representing hypoxic tissue from a previous study in HNC, where this level was determined based on a voxelwise comparison between perfusion and uptake of [^18^F]EF5 in tumour [7]. The median FHV of the tumours (20.2%) in the present study was slightly smaller compared to some previous studies with [^18^F]FMISO and [^18^F]FETNIM PET/CT using MATV-based GTV delineation in HNC patients [19]. However, any threshold for hypoxia is an estimation reflecting the present patient population and applied methodology and instrumentation. Therefore, correlation and agreement between the repeated scans across the whole scale of uptake rates including “non-hypoxic” areas are essential for correct judgement in a test-retest study.

We found that tumour-level parameters (SUVmean, SUVmax, and TMR) showed a high correlation and repeatability between the paired [^18^F]EF5 scans. These results agree with the previous studies in HNC and lung cancer using hypoxia-activated 2-nitroimidazole tracers [^18^F]FMISO [10, 11] and [^18^F]HX4 [12]. However, the oldest study using [^18^F]FMISO PET/CT [9] reported a lower correlation between the repeated scans compared to ours and the above-mentioned three studies [10–12]. Several explanations for this controversy have been proposed, including an inconsistent uptake time within the repeated scans, the use of either 2D or 3D acquisition modes, and the variability of image co-registration algorithms [11]. The results of the present study support the perception of the need for highly consistent imaging protocol and data processing algorithms to be applied in analyses addressing the repeatability of hypoxia PET studies.

The voxel-by-voxel analysis showed good or moderate spatial correlation in [^18^F]EF5 uptake between the paired scans. Using *r* > 0.5 as a level of strong correlation and reproducible results similarly as in two previous studies [9, 12], we observed a strong voxelwise correlation for 9 out of 10 of our patients. On the other hand, a slightly lower agreement using relative mean difference and relative CoR between the voxel uptake of paired scans was observed in this study compared to those of Grkovski et al. [11] and Zegers et al. [12]. Nevertheless, comparing results between the present and all previous [9–12] studies is challenging due to some heterogeneities in acquisition parameters. A crucial parameter affecting voxel-level repeatability is the used voxel size in images which was not reported by Okamoto et al. [10] and Zegers et al. [12]. We used a voxel size of 3.65 × 3.65 × 3.27 mm, similar to our institutional diagnostic protocol and representing the high end of the reported resolutions used in other corresponding studies [9, 11]. Another parameter not uniformly available for comparison is the tumour size [10], which has an influence on partial volume effect. However, the distribution of tumour size in our study seems to be comparable to those reported in previous studies of HNC [9, 12]. Finally, special attention should be given to statistical methods to address repeatability in a test-retest design, and following this, we calculated both correlation and agreement values for all uptake parameters [20].

There is clearly a trend of larger variability in tumour hypoxia when a longer time period is assessed, although limited data on temporal changes is available with hypoxia detection methods other than PET [21]. Our previous preclinical PET/CT study showed a large variation in intratumour uptake of [^18^F]EF5 in xenografted HNC at different stages of tumour growth up to 5–36 days apart [22]. On the other hand, preclinical studies using a short interval from 6 h to 1 day have reported a high repeatability of hypoxia imaging with [^18^F]FAZA PET [23] and [^18^F]FMISO PET [24]. In the present study, the median time of 7 days between the repeated scans was longer than that in several previous clinical studies that reported an average interval of 1–3 days [9–12, 25]. However, we did not observe any trend for lower repeatability of tracer uptake parameters compared to those studies where interscan time was shorter. Consequently, we consider that the impact of the difference between the intervals of the present and previous studies is small.

This study also has some limitations. In line with previous studies [9–12], the number of patients was reasonably small. In addition, all of the study subjects were men. These limitations derived from the challenges in subject enrollment as well as the remarkably higher incidence of pharyngeal cancer among male patients. Partial volume effect might increase the variability of tracer uptake between paired scans, especially among small and irregularly shaped tumours. Finally, the repeated setup of patients as well as the co-registration of images are known to be prone to some kind of inaccuracies, although optimal methods and head and neck immobilisation masks are used [26]. Nevertheless, these issues that may cause a decrease in the repeatability of tracer uptake are pragmatic challenges, present in everyday clinical imaging and image analysis.

The feasibility of [^18^F]EF5 PET/CT for guiding RT dose escalation or adaptation deserves attention in the future. The pretreatment hypoxia-specific signal of [^18^F]EF5 at 3 h from injection in HNC is repeatable and comparable to those of [^18^F]FMISO and [^18^F]HX4 at 4 and 2 h, respectively [10, 12]. Given the similarities in the chemical properties of the three tracers, this is not surprising, while small differences in their sensitivities to acute vs. chronic hypoxia may occur [27]. Recently, a few clinical studies were performed where changes in tumour hypoxia defined with PET/CT were monitored during the first weeks of RT. In general, these studies state that residual hypoxia after the first week or two of RT is more stable and shows more prognostic significance compared to pretreatment tumour hypoxia [28, 29]. Thus, an important future study should investigate the stability and prognostic significance of [^18^F]EF5 uptake during the course of RT. Another central clinical point of view to be assessed is whether [^18^F]EF5 PET/CT qualifies for the selection of patients for hypoxia-targeted interventions, such as treatment with hypoxia-avid radiosensitisers or hypoxia-activated prodrugs [30].

## Conclusion

A high repeatability of tumour-level tracer uptake was observed in the paired [^18^F]EF5 PET/CT scans acquired before the onset of CRT. The voxel-by-voxel analysis showed predominantly good correlation and agreement between the repeated scans. We thus encourage further evaluation of [^18^F]EF5 PET/CT for guiding hypoxia-targeted treatment interventions.
